# The contribution of an asthma diagnostic consultation service in obtaining an accurate asthma diagnosis for primary care patients: results of a real-life study

**DOI:** 10.1038/s41533-017-0027-9

**Published:** 2017-05-19

**Authors:** R. M. E. Gillis, W. van Litsenburg, R. H. van Balkom, J. W. Muris, F. W. Smeenk

**Affiliations:** 1 Department of Pulmonology, Catharina Hospital Eindhoven, Eindhoven, Netherlands; 2 Department of Family Medicine, Research institute CAPHRI, Maastricht University Medical Centre, Maastricht, Netherlands

## Abstract

Previous studies showed that general practitioners have problems in diagnosing asthma accurately, resulting in both under and overdiagnosis. To support general practitioners in their diagnostic process, an asthma diagnostic consultation service was set up. We evaluated the performance of this asthma diagnostic consultation service by analysing the (dis)concordance between the general practitioners working hypotheses and the asthma diagnostic consultation service diagnoses and possible consequences this had on the patients’ pharmacotherapy. In total 659 patients were included in this study. At this service the patients’ medical history was taken and a physical examination and a histamine challenge test were carried out. We compared the general practitioners working hypotheses with the asthma diagnostic consultation service diagnoses and the change in medication that was incurred. In 52% (*n* = 340) an asthma diagnosis was excluded. The diagnosis was confirmed in 42% (*n* = 275). Furthermore, chronic rhinitis was diagnosed in 40% (*n* = 261) of the patients whereas this was noted in 25% (*n* = 163) by their general practitioner. The adjusted diagnosis resulted in a change of medication for more than half of all patients. In 10% (*n* = 63) medication was started because of a new asthma diagnosis. The ‘one-stop-shop’ principle was met with 53% of patients and 91% (*n* = 599) were referred back to their general practitioner, mostly within 6 months. Only 6% (*n* = 41) remained under control of the asthma diagnostic consultation service because of severe unstable asthma. In conclusion, the asthma diagnostic consultation service helped general practitioners significantly in setting accurate diagnoses for their patients with an asthma hypothesis. This may contribute to diminish the problem of over and underdiagnosis and may result in more appropriate treatment regimens.

## Asthma: Service helps general practitioners make accurate diagnoses

A consultation service can help general practitioners more accurately diagnose asthma and select the appropriate treatments for their patients. Researchers in The Netherlands, led by Frank Smeenk from Catharina Hospital in Eindhoven, describe an asthma diagnostic consultation service they created to support GPs in their diagnostic process for patients suspected of having asthma. Over a four-year period, the service received a total of 659 referrals and only confirmed the diagnosis of asthma in 275 cases. Another 20 patients had asthma overlapping with chronic obstructive pulmonary syndrome. The service also picked up other diseases, such as rhinitis, that general practitioners had missed. Overall, because of the consultation service and its revised diagnoses, more than half of all patients adjusted their medications. Most patients required only a single consultation and could then be referred back to their physicians.

## Introduction

Previous studies showed that it may be difficult to make an accurate diagnosis of asthma.^1–9^ This can result in both under and overdiagnosis of asthma. A recent review by José et al. showed that the lack in precision for asthma in primary health units ranged from 54% underdiagnosis to 34% overdiagnosis.^10^ Underdiagnosis can result in increased morbidity and mortality.^11^ Conversely, overdiagnosis may result in unnecessary treatment with unnecessary possible side effects of medication, and higher costs. Several studies in the Netherlands emphasised this diagnostic uncertainties.^2–4^ One study in the Netherlands estimated that 30% of all patients with respiratory complaints used inhaled corticosteroids (ICS) without a clear indication and that more than 10% of all these patients used these drugs unnecessarily.^3^

To diagnose asthma in patients, general practitioners (GPs) in the Netherlands use a national guideline.^12^ According to this guideline an asthma diagnosis is a clinical one based on history, physical examination and preferably a confirmatory spirometry. A spirometry is considered to be confirmative when it shows a significant bronchodilatation after inhalation of a bronchodilator (i.e., an increase in Forced Expiratory Volume in 1 second (FEV_1_) of more than 12% and more than 200 mL compared to the FEV_1_ before inhalation of the bronchodilator).^12^ Because most stable asthma patients will have a normal spirometry, a confirmatory spirometry will be hard to get (i.e., at baseline these patients do not have a broncho-obstruction, therefore demonstration of a significant bronchodilatation will be virtually impossible).^12, 13^ On the other hand some asthmatics may have a more persistent airway inflammation because of inflamed oedematous bronchial walls and/or chronic asthma may have resulted in ‘airway-remodelling’. Both conditions will be unresponsive to bronchodilatory medication. This may lead to a ‘negative’ reversibility test.^11–13^ Because of this seemingly irreversible character of the obstruction in these patients, they might be misclassified as having chronic obstructive pulmonary disease (COPD). So, in terms of diagnostics these two categories of asthma patients will pose diagnostic problems for GPs, despite their detailed guideline description.

To tackle this problem we installed an ‘asthma diagnostic consultation service’ (ADCS) for GPs in our region. This is a ‘one-stop-shop’ outdoor policlinic at the Pulmonology Department of the Catharina Hospital in Eindhoven, the Netherlands.

During this visit the medical history is examined and a physical examination as well as a histamine challenge test (HCT) are performed.

In this study we evaluated the usefulness and performance of this ADCS over the last 4 years with the following research questions:

1. What is the (dis)concordance between the GPs working hypotheses and the ADCS diagnoses and the possible consequences this might have had on pharmacological therapy?
2. How many patients were referred back to the GP after one visit and if further follow-up was necessary by the ADCS, what were the reasons and how long did this last?

## Results

### Patients

Over a period of 4 years, 174 GPs referred a total of 659 patients to the ADCS. The mean age was 45.3 years (range 13–85) and 40% was male (*n* = 266) and 60% was female (*n* = 393).

### Diagnostic process at the ADCS

**Fig. 1:**
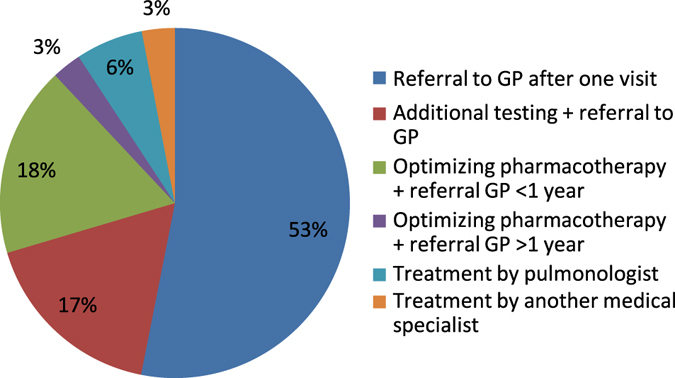
Follow up approach after the first consultation

Three hundred fifty patients (53%) were referred back to their GP after one visit and 114 patients (17%) after additional diagnostic tests were conducted (Fig. 1). These could be a chest X-ray, HRCT, bodybox, phadiatop, CT sinuses, bronchoscopy, maximal exercise test, ECG, polysomnography, or a hyperventilation provocation test. In cases of a possible non-pulmonary diagnosis, patients were referred to another specialist. (see online figure for distribution Figure e1).

### Distribution of the various diagnoses and concordance between GPs working hypotheses and diagnoses recorded at the ADCS

#### Working hypotheses by the GP

The 659 patients who were referred to the ADCS had the following GP working hypotheses: possible asthma (based on the Nederlands Huisartsen Genootschap (NHG)-guideline) in 644 patients (97%), asthma COPD overlap syndrome (ACOS) in 10 patients (2%) or COPD in 4 patients (1%) (Table 1). In 189 out of 644 patients with the working hypothesis of asthma, patients were treated by their GP with ICS.

**Table 1:** GP’s working hypotheses vs. diagnoses recorded at the ADCS

		Diagnoses recorded at the ADCS					
		No asthma	Asthma	COPD	ACOS	Other pulmonary diagnosis	Total
GP’s working hypotheses	No asthma	0	0	0	0	0	0
	Asthma	338	270	14	13	9	644
	COPD	1	2	0	1	0	4
	ACOS	0	3	1	6	0	10
	Other pulmonary diagnosis	1	0	0	0	0	1
	Total	340	275	15	20	9	659

Concordance working hypotheses GP and diagnoses recorded at ADCS: 42%

#### Definitive diagnosis at the ADCS

In 340 of all referred patients (52%) an asthma diagnosis was excluded, whereas the diagnosis was confirmed in 275 patients (42%). Twenty patients (3%) were diagnosed having ACOS. (Table 1). In a subgroup analysis of the patients aged between 12–18 years of age asthma was excluded in 6 patients and confirmed in 15 patients.

### Concordance between GPs working hypothesis and ADCS diagnosis

Table 1 displays the (dis)concordance between the GPs’ working hypotheses and diagnoses recorded at the ADCS. Overall concordance was only 42%.

### Comorbidity in asthma patients

One hundred thirty-seven patients diagnosed with asthma were also diagnosed with rhinitis (50%) and 11 patients (4%) with gastro-oesophageal reflux disease (GERD) by the ADCS (Table 2).

**Table 2:** Other non-pulmonary diagnoses recorded at the ADCS

ADCS Diagnoses	Rhinitis	GERD	Post infectious cough	Smoking related cough	Other
No asthma (*n* = 340)	112	58	59	15	39
Asthma (*n* = 275)	137	11	11	3	6
ACOS (*n* = 20)	8	1	0	0	0

### Primary non-asthma diagnoses

Other non-pulmonary diagnoses responsible for the patient’s symptoms when asthma was excluded were rhinitis (*n* = 99), GERD (*n* = 55), post-infectious cough (*n* = 54), smoking related cough (*n* = 15) or another non-pulmonary diagnosis (*n* = 39) (Table 2).

### Concordance between non-pulmonary diagnoses recorded by the GP and ADCS

Of all referred patients, the non-pulmonary diagnoses made *by GPs* included chronic (non) allergic rhinitis (*n* = 163), GERD (*n* = 1), post-infectious cough (*n* = 1) or, in the case of one patient, an obstructive sleep apnoea syndrome. In total, non-pulmonary diagnoses made at *the ADCS* included chronic (non) allergic rhinitis (*n* = 261), GERD (*n* = 71), post-infectious cough (*n* = 71), smoking related cough (*n* = 19), medication related cough (*n* = 6) or another non-pulmonary diagnosis (*n* = 45).

One hundred and six patients (16%) were not diagnosed with rhinitis by their GP while this was noted at the ADCS (Table 3).

**Table 3:** Concordance between chronic rhinitis diagnosed by the GP vs. ADCS

	ADCS rhinitis: No	ADCS rhinitis: Yes	Total
GP Rhinitis: No	390	106	496
GP Rhinitis: Yes	8	155	163
Total	398	261	659

Cohen’s *κ* = 0.613

### Consequences of adjusted ADCS diagnosis for therapy

**Fig. 2:**
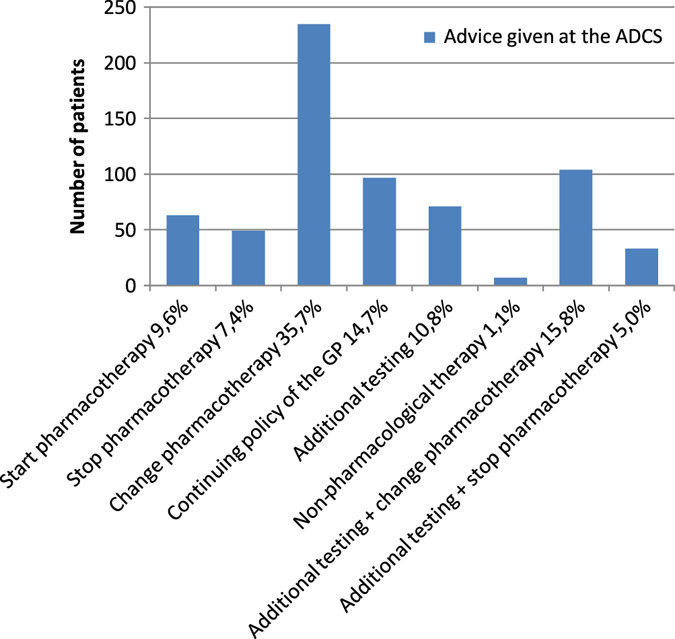
Advice given at the ADCS. The *bars* on the *x*—axis indicate the advice given by the ADCS after consultation. The *y*—axis represents the numbers of patients

The adjusted ADCS diagnosis resulted in a change in pharmacotherapy in 74% of all patients: 63 patients (10%) received the advice to start pharmacotherapy, 339 patients (51%) to change pharmacotherapy and 82 patients (12%) to stop pharmacotherapy. For 168 patients (26%) the medication of the GP was unchanged (Fig. 2).

Concerning the individual classes of inhalers, 178 patients (27%) received the advice to start an ICS, 151 patients (23%) to start ICS with a LABA and 12 patients (2%) to start with a Long-Acting Anticholinergics (LAMA). Seventy-five patients (11%) received the advice to stop an ICS, 56 patients (8%) to stop LABA and 13 patients (2%) to stop with Short-Acting Anticholinergics (SAMA).

**Fig. 3:**
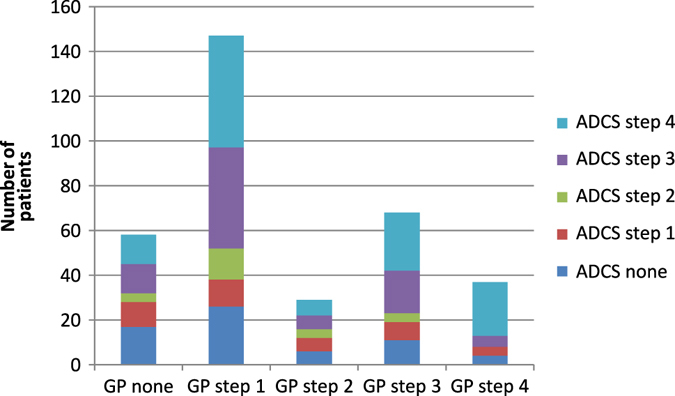
Stepwise approach to asthma treatment: GP vs. ADCS (*n* = 340). The *bars* on the x—axis indicate the numbers of patients being set on no medication or a GINA step 1–4 treatment by their GP. The various *colours* in the *bars* represent the proportion of patients being set on no or GINA step 1–4 treatment by the ADCS after consultation

In case of a stable or unstable asthma, the patient’s medication was adjusted according to the stepwise approach as described in the NHG and Global Initiative for Asthma (GINA) guidelines. The way this was done is depicted in Fig. 3. Stepping up was the most frequently given advice by the pulmonologist.

### Duration of the follow-up at the ADCS

Three hundred fifty patients were referred back to their GP after just one visit: for 116 patients (18%) the medication was optimised and after stabilisation of their asthma they were referred back within 1 year. For only 18 patients (3%) optimising the therapy took more than 1 year before they could be referred back (Fig. 1). Forty-one patients (6%) are still under control at the ADCS because of a severe unstable asthma and 20 patients (3%) were referred to another medical specialist because of a primary non-pulmonary diagnosis (Fig. 1)

## Discussion

### Main findings

This study showed that the GPs who referred their patients to our ADCS rightly had suspicion about their asthma working hypothesis.

In 52% of all referred patients an asthma diagnosis could be excluded. This is in line with the findings of Lucas et al. who found that in only about half of all patients in primary care with suspected asthma, an asthma diagnosis was confirmed.^4^ A possible explanation for the GPs overdiagnosis of asthma is that the urge to treat the patient’s symptoms possibly related to asthma and to start an ICS, might be sometimes greater than completing the diagnostic process first.

Furthermore, the adjusted diagnosis lead to a change in pharmacotherapy in 74% of the patients referred to the ADCS, whereas in 10% of the cases pharmacotherapy was started and for 12% all respiratory medication could be stopped.

The discrepancy found in the frequency of other (non-pulmonary) diagnoses made by GPs vs. the ADCS, mainly concerning chronic rhinitis and GERD, was remarkable. The GPs diagnosed chronic rhinitis in 163 patients, whereas this was set by the ADCS in 261 patients, i.e., an increase of 62%. GERD was only noted in 1 patient by her GP and in 71 patients by the ADCS.

Most patients were referred back to their GP after only one consultation and nearly all patients within 6 months, indicating that the intention of this one stop shop policy was achieved in most patients. Only 6% of all patients is still under control at the ADCS because of a severe unstable, brittle asthma.

In conclusion, the ADCS helped GPs significantly in setting an accurate diagnoses in their patients in whom they had some uncertainty about their asthma working hypothesis. This resulted in a change of their maintenance medication in the majority of patients.

### Comparison with existing literature

Multiple studies showed that GPs in various different health care settings find it difficult to set an accurate diagnosis of asthma.^1–9^ According to a review article this can result in both under (as much as 54%) and overdiagnosis (till 34%) of asthma.^10^ Our study illustrates the limitations of a GPs working hypothesis of asthma which is in line with previous studies.^1–9^ A previous study in the Netherlands estimated that more than 10% and maybe even up to 30% of all patients with respiratory problems (both COPD and asthma patients) in primary care used ICS unnecessarily.^3^ Our findings are in line with these results as 11% of all our patients received the advice to stop their ICS. On the other hand, 27% of all patients received the advice to start ICS, illustrating the problem of under-treatment. A previous study in Denmark even showed an under-treatment of asthma of 76%.^14^

The prevalence of ACOS among asthma patients in our study-population (6%) is lower than reported in previous studies showing a prevalence range from 13 to 61% of ACOS among patients with asthma.^15^ This difference might be explained because of the different populations studied in the various studies. Furthermore, our service was an *asthma* and not a *COPD* consultation service.

### Other non-pulmonary diagnoses

Our study also demonstrates that in primary care the attention for rhinogenic complaints in asthma patients might be improved. Various studies showed that a significant proportion of patients with both allergic and non-allergic asthma also have rhinitis and optimising rhinitis treatment is also an important issue in asthma treatment.^16, 17^ This is in line with our study illustrating that 50% (*n* = 137) of all asthmatics were also diagnosed with rhinitis. Vice versa, 10–40% of all patients with allergic rhinitis have asthma.^17^

This under-diagnosis of rhinitis in asthmatics in primary care is now being acknowledged in the recently (2015) updated version of the Dutch NHG guideline on asthma.^12^ Further implementation of this guideline might tackle this problem. The internationally used GINA guideline also highlights the importance of an early diagnosis of rhinitis for asthma patients.^13^

Another remarkable difference in non-pulmonary diagnosis made by the GP and ADCS was GERD (*N* = 1 vs. *N* = 71). Although GERD is an important comorbid condition in asthmatics, this high percentage of GERD found in our population might also be explained by the large number of patients who had chronic cough as their primary complaint (15%).^18^ GERD is an important common cause of chronic cough.^19^

### Limitations of the study

Our study population consisted of patients referred by the GPs, primarily to set or exclude an asthma diagnosis. Thus, our study population is a selection of patients in primary care in which GPs most probably have had more problems in obtaining an accurate diagnosis of asthma. So, whether or not our results might be extrapolated to all patients suspected to have asthma in whom the GP does not consider referral might be questionable. Considering the results of Lucas et al. and Jose et al. showing a considerable percentage of overdiagnosis in *all* primary care patients with respiratory complaints, future prospective research should clarify this.^3, 10^

Finally, an important limitation in our study is that only patient data from one region were analysed. Previous research has shown that in this region the attention to asthma and COPD by GPs is high.^2, 4^ Thereby it might be assumed that the knowledge of GPs of asthma/COPD in our region might be higher than average. For this reason, it might also be possible that our result reflects an underestimation of the real problem.

## Conclusion

In conclusion the ADCS helped GPs significantly in setting an accurate diagnoses in their patients with an asthma working hypothesis. This may contribute to diminish the problem of over and underdiagnosis and may result in more appropriate treatment regimens.

## Methods

### Subjects

In this retrospective study we included all patients (>12 years) who were referred by their GP to the ADCS at the Catharina Hospital Eindhoven, the Netherlands, from May 2011 until August 2015 with the suspicion of possible asthma. The Catharina Hospital is a large (750 bed) teaching hospital in the South of the Netherlands.

The GPs working hypothesis of asthma was, according to National guidelines,^12^ based on medical history, physical examination and a spirometry with reversibility testing according to American Thoracic Society/European Respiratory Society (ERS) guidelines.^11, 13, 20^

We registered demographics (age and gender), the reason for referral, the GPs working hypotheses, the possible secondary (non-pulmonary) diagnoses indicated by GPs and the patient’s medication.

Concerning medication, we focused on the various drug classes used in asthma and chronic rhinitis being Short-Acting Beta-2-Agonists, ICS, Long-Acting Beta-2-Agonists (LABA), SAMA, LAMA, Antihistamines, and Nasal Corticosteroids.

### Diagnostic process of the ADCS

The GPs provided their patients with information about the HCT and instructed them (if applicable) to stop antihistaminic drugs 7 days before the test and to discontinue inhaled bronchodilators for at least 12 till 84 h before the test according to the half life time of their inhalation medication.

At the ADCS the patients’ medical history was taken and a physical examination and HCT were carried out. In case of a normal HCT, symptomatic asthma can reliably be excluded, for the negative predictive value of this test is close to 100%. If the histamine threshold (HT) is lower than 1 mg/ml with a compatible medical history, the diagnosis of asthma can reliably be made as the positive predictive value in this case is nearly 100%.^21^ In cases of a HT between these values, the diagnosis may be uncertain and depends on the compatibility of medical history with the HT, recognition of complaints by the patient during the test and the patient’s responsiveness to treatment.^21^ The HCT was performed by a certificated lung function analyst according to a standardised protocol based on ERS standard procedures.^20^

An asthma diagnosis was considered to be confirmed in case of a compatible medical history, with a confirmative HCT.

Additional tests might be performed if other diagnoses than asthma were considered. In case asthma was confirmed, patients were treated according to the national guideline of the Dutch College of Family Physicians for asthma which is comparable with the GINA guideline.^13^

### Diagnostic and therapeutic work-flow at the ADCS after the first visit

The ADCS was intended to be a ‘one-stop-shop’ outdoor policlinic. However, if the patient’s asthma was unstable or if no final confident diagnosis could be made in this consultation, patients were followed up for more than one consultation. Several reasons could be identified:

1. No definitive diagnosis could be made at the first consultation. Additional tests were necessary after which patients were referred back to their GPs with a final diagnosis and therapeutic advice.
2. Asthma diagnosis was confirmed, but due to severe instability (non)pharmacotherapeutical treatment had to be optimised. After stabilisation, patients were referred back to the GPs with therapeutic advice within 1 year after consultation.
3. Asthma diagnosis was confirmed but specialist care was needed for more than 1 year because of severe unstable asthma.
4. Another pulmonary diagnosis was made for which continued care by the pulmonologist was necessary.
5. Another non-pulmonary diagnosis was made for which continued specialist care was necessary.

### Evaluation of performance of the ADCS

To evaluate the performance of the ADCS we examined the concordance between the GPs’ working hypotheses and the final diagnoses recorded at the ADCS.

The non-pulmonary diagnoses included chronic rhinitis, GERD, post-infectious cough, smoking-related cough (active smokers who cough in whom other causes of cough were excluded) or other non-pulmonary diagnosis. These diagnoses were based on clinical grounds with evaluation of symptoms after a trial with appropriate medication.

Lastly we examined the concordance between the pharmacotherapy based on respiratory symptoms prescribed by the GP and the advice given at the ADCS.

### Data analysis

The statistical package SPSS 21 was used to analyse the data.

For the description of nominal variables we used frequency tables. Crosstabs were used for analysing and comparing the results as discussed before. Continuous variables were described in terms of means, standard deviations and confidence intervals.

## Electronic supplementary material

Figure e1
