# Supplementary figures and images for: The contribution of an asthma diagnostic consultation service in obtaining an accurate asthma diagnosis for primary care patients: results of a real-life study
Source: NPJ Prim Care Respir Med. 2017 May 19;27:35. doi: 10.1038/s41533-017-0027-9 (PMC5438345; doi:10.1038/s41533-017-0027-9)

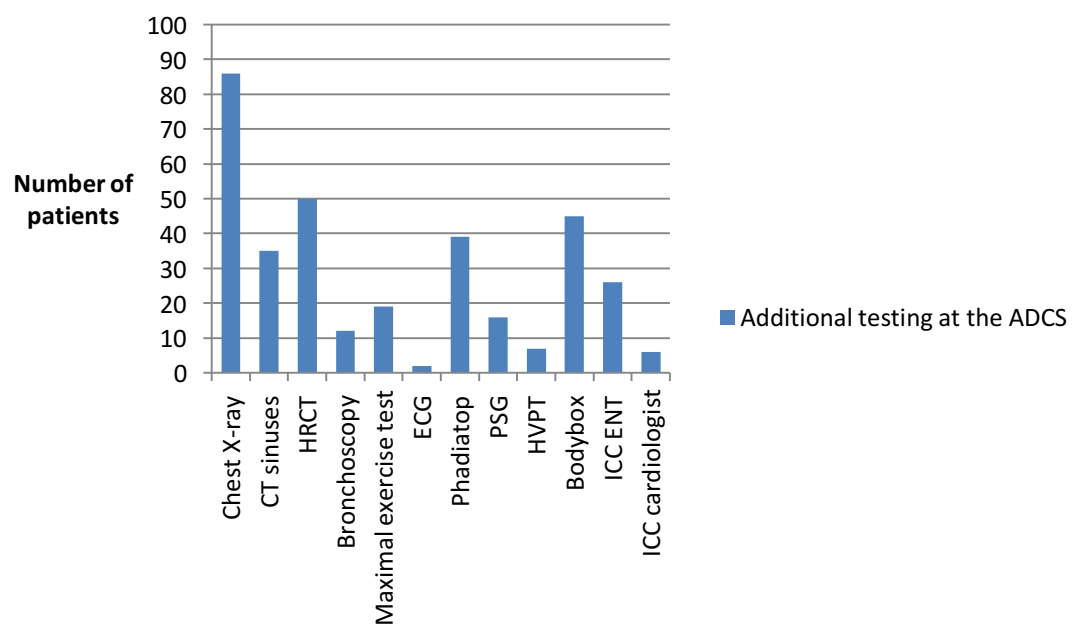

**Figure e1 (extra for online publication)** Additional testing at the ADCS

Supplement: Supplementary file 1 — Figure e1 [file 41533_2017_27_MOESM1_ESM.pdf]
